# First report of the genus *Kisaura* Ross (Trichoptera, Philopotamidae) from India with the description of six new species

**DOI:** 10.3897/zookeys.152.1125

**Published:** 2011-12-08

**Authors:** Manpreet Singh Pandher, Malkiat Singh Saini

**Affiliations:** 1 Department of Zoology and Environmental Sciences, Punjabi University, Patiala, India-147002

**Keywords:** Trichoptera, Philopotamidae, Philopotaminae, systematic, *Kisaura*, *Sortosa*, new species, Oriental Region, India, male genitalia

## Abstract

The genus *Kisaura* Ross, 1956 (Trichoptera, Philopotamidae, Philopotaminae) is reported from India the first time. Six new species are described and illustrated: *Kisaura elongata* **sp. n.**, *Kisaura eloct* **sp. n.**, *Kisaura clavata* **sp. n.**, *Kisaura gangtokensis* **sp. n.**, *Kisaura truncata* **sp. n.**, all from Gangtok (Sikkim) and *Kisaura himachalica* **sp. n.** from Barot (Himachal Pradesh). Male genitalia of this genus are distinguishable from those of other genera of the family by the pair of long lateral processes of tergum X, the well-developed mesoventral plates between two segments of the inferior appendages, and by the brush-like row of dark setae on the inner surfaces of the terminal segments (harpago) of the inferior appendages.

## Introduction

*Kisaura* was established by Ross (1956) as a subgenus of *Sortosa* Navás (1918), based on *Sortosa obrussa* Ross (1956) as its type species. It was considered a subgenus of *Dolophilodes* Ulmer (1909) by Kuhara (1999), based on the precedence of the generic name *Dolophilodes* over that of *Sortosa* as pointed out by Ulmer (1957). *Sortosa* Navás (1918) is not a synonym of *Dolophilodes* Ulmer (1909) as indicated by Schmid (1964) by writing “*Dolophilodes* (*Sortosa*).” *Kisaura* was subsequently raised to the status of an independent genus by Sun and Malicky (2002), based on the typical and distinct male genitalic structures. *Kisaura* isthought to have originated in the Oriental Region where the greatest number and most primitive of its species occur (Ross 1956).

This genus is currently represented by 42 species, mostly confined to the Oriental and Palaearctic Regions (Morse 2011). Twenty-seven species (more than ½) in the genus occur in the Oriental Region, many of which were transferred from *Dolophilodes* Ulmer. Most of the recent additions to *Kisaura* were made by Malicky and co-workers (Malicky 1993a, 1993b, Malicky and Chantaramongkol 1993a, 1993b, Malicky 1995, Sun and Malicky 2002, Malicky 2007, Malicky 2009), who added 17 new species to this genus from Thailand, Bhutan, China, and Vietnam. The genus *Kisaura* is reported from India for the first time here, with the description of six new species.

Ross 1956 divided the species of *Kisaura* into two major groups, a primitive one from Myanmar with species in which the basal segment (coxopodite) of inferior appendage is longer than the apical segment (harpago), and another more specialized group from Southeast China, Japan, and Russian Far East in which the basal segment of the inferior appendage is shorter than the apical segment. Based on current diversity, there is inconsistency in the species groups originally defined by Ross (1956), presumably because they were based on very few known species. In addition to the characters specified by Ross (1956), species can be distinguished also by the variation in the shape and length of lateral spiniform processes of tergum X and the black comb-like setae on apical segment of inferior appendage. The biology of the species in the genus is poorly known (Hur and Morse 2006). A more thorough study and complete diagnosis of all previously known and newly described species will be required, along with a study of the larval stages and a well supported phylogenetic analysis, to understand the origin and dispersal of *Kisaura* to other parts of Eastern Asia.

## Materials and methods

Adults were collected by light traps (mercury vapour bulb and UV) placed near the edge of high altitude streams of the Himalayan belt of India. The species described here were mainly collected from Gangtok (Sikkim) in very dense humid forests at altitudes ranging from 1800 m to 2100 m. One species was collected from Himachal Pradesh. The specimens were preserved in 70% ethyl alcohol with a drop of glycerol added. Pertinent collection and locality data were recorded.

For species level identification it is essential to observe the lateral processes of the Xth tergite which are hidden below the preanal appendages in lateral view and are also not clearly visible even in dorsal view. For accomplishing this, the male genitalia were removed from the specimens and put in 10% KOH solution overnight. After this treatment the genitalia were put in 80% ethyl alcohol with a drop of glycerol and observed for morphological characters. The drawings of various aspects were done with the aid of zoom stereoscopic binocular microscope (with maximum magnification of 120×) fitted with an ocular grid in one eye piece. The final drawings were rendered in black ink. The illustrations were scanned at 600 dpi grayscale, and mounted onto plates in Adobe© Photoshop© 7.0. The genitalic terminology corresponds to Ross (1956) and Hur and Morse (2006).Type specimens are deposited in the Punjabi University Patiala Museum (PUPM), Department of Zoology and Environmental Sciences, Punjabi University, Patiala. Additional material examined in also listed, but these specimens were damaged and considered of poor quality to include as paratypes.

## Systematics

### Genus Kisaura

Ross, 1956

http://species-id.net/wiki/Kisaura

#### Type species.

*Sortosa obrussa* Ross 1956: 57 (original designation).

#### Description.

Spurs: 2, 4, 4; wings with primitive venation except fork I variable: it may be near or considerably beyond sectorial cross vein s, or R_2 _may be atrophied and 2A of forewing incomplete (Ross 1956). Male genitalia with pair of lateral processes between Xth tergite and preanal appendages; inferior appendages simple, with mesoventral plate developed between two segments; apical segment of inferior appendage with diagnostic longitudinal row of spine-like setae on its inside mesal surface.

#### Distribution.

Oriental and Palaearctic Regions.

#### Diagnosis.

The genus *Kisaura* can be easily separated from *Dolophilodes* Ulmer by a pair of elongate and sclerotized lateral processes of segment X and by the brush-like row of dark setae on the inner surface of apical segment of the inferior appendages which are lacking in *Dolophilodes*.

### Species descriptions

#### Kisaura clavata

Pandher & Saini sp. n.

urn:lsid:zoobank.org:act:CA276C7B-013B-4C7C-A428-961110C17518

http://species-id.net/wiki/Kisaura_clavata

Figs 1–5 

##### Description.

In superficial comparison, this species seems somewhat allied to *Kisaura moselyi* Kimmins, 1955 from North East Myanmar, but the combination of characters, including segment VIII with a deep median V-shaped indentation, clavate preanal appendages, and basal part of the apical segment of the inferior appendage with a stout spine like setae, sets *Kisaura clavata* sp. n. apart from *Kisaura moselyi*.

Adult. Color in alcohol entirely fulvous except mesoscutellum flavid; maxillary palp with pale annulations at the joints; head with golden and fulvous pubescence; antenna moderately long, scape:pedicel ratio = 1.66: 1; maxillary palp segments ratios 1: 2: 3: 4: 5 = 1: 2.4: 2.6 : 2.6: 5.3; labial palp segments ratios 1: 2: 3 = 1: 1.75: 3.1. Length of forewing 7 mm, sprinkled with white patches along the posterior margin and covered with moderate, sparse and brownish setae, pterostigma not prominent in both wings; discoidal cell very small in forewing; fork I absent in both wing.

**Figures 1–5.**
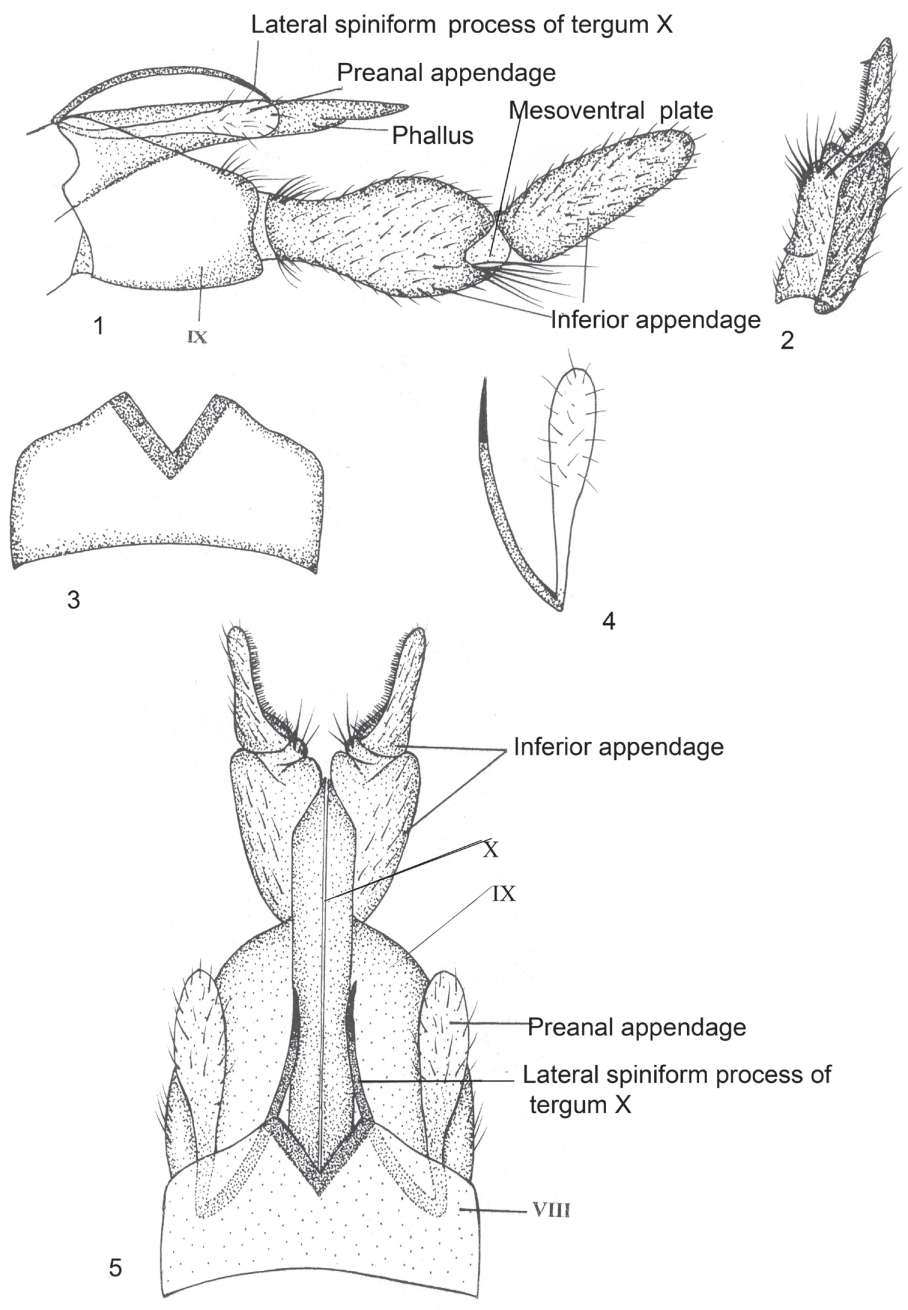
*Kisaura clavata* sp. n. **1** Male genitalia, lateral **2** Inferior appendage, dorsal **3** Tergite VIII, dorsal **4** Preanal appendage, dorsal **5** Male genitalia, dorsal. Abbreviations: VIII, IX, X = abdominal segments VIII, IX, and X, respectively

Male Genitalia (Figs 1–5). Sternum VIII without ventral process; tergum VIII with deep median V-shaped zone of spines. Segment IX rather short, sclerotized, quadrate with median prominence on anterolateral side, posterolaterally with shallow, rounded excision, apically setose. Inferior appendages two-segmented; basal segment (coxopodite) little longer and stouter than apical segment (harpago), narrow at its base, broad and rounded towards apex, in lateral view, with two lobes, inferior lobe with tuft of long setae; apical segment with curved row of dark brush-like setae and stout spine-like setae at base on mesal surface in dorsal view. Tergum X membranous, extending beyond apex of segment IX, at base on each side arises blade-like, lateral spiniform process, with articulated spinelet at apex, recurved cephalad and then caudoventrad, reaching up to apex of segment IX. Preanal appendage as long as spiniform process of tergum X and clavate at apex. Phallus membranous, intimately surrounded by tergum X.

**Holotype male: INDIA: Sikkim: Gangtok:** 27°36'0"N, 88°37'0"E, 1700 m, 25.v.1999, M. Saini, (PUPM).

##### Paratypes.

same data as holotype, 2 males (PUPM).

##### Additional material.

 **INDIA: Sikkim: Gangtok**: 27°36'0"N, 88°37'0"E, 1,700m, 15.v.2011, Parey, 1 male (PUPM).

##### Distribution.

India: Sikkim.

##### Etymology.

Because of the club-like, (i.e., clavate) apex of preanal appendage, this species has been named *clavata*.

#### Kisaura elongata

Pandher & Saini sp. n.

urn:lsid:zoobank.org:act:B6A4576F-56E1-4811-A60F-031FF8F3535C

http://species-id.net/wiki/Kisaura_elongata

Figs 6–10 

##### Description.

This species is allied to *Kisaura sura* Malicky & Chantaramongkol, 1993 and *Kisaura consagia* Malicky & Chantaramongkol, 1993, both reported from Thailand, because of the presence of bilobed preanal appendages. However, *Kisaura elongata* is sufficiently distinct from these two species as the two lobes are unequal, i.e., one lobe of the preanal appendage is long while the second is very small, and also in having elongate and inwardly recurved lateral spiniform process of tergum X. In *Kisaura sura* and *Kisaura consagia* the two lobes of preanal appendage are almost equal and the lateral spiniform process of tergum X is not inwardly recurved.

Adult. Color in alcohol entirely fulvous excepting antenna with dark brown annulation at joints; body covered with moderate, sparse and fuscous setae excepting head with nigrescent pubescence. Antenna almost equal to length of forewing; scape:pedicel ratio = 1: 0.58; maxillary palp segments ratios 1: 2: 3: 4: 5 = 1: 2: 2.6: 1.6: 4.3 and labial palps segments ratios 1: 2: 3 = 1: 0.8: 1.8. Length of forewing 6 mm, sprinkled with yellow patches, in both wings pterostigma not prominent; discoidal cell almost triangular; apical fork I present in both wings, in forewing positioned considerably beyond sectorial cross-vein s.

**Figures 6–10.**
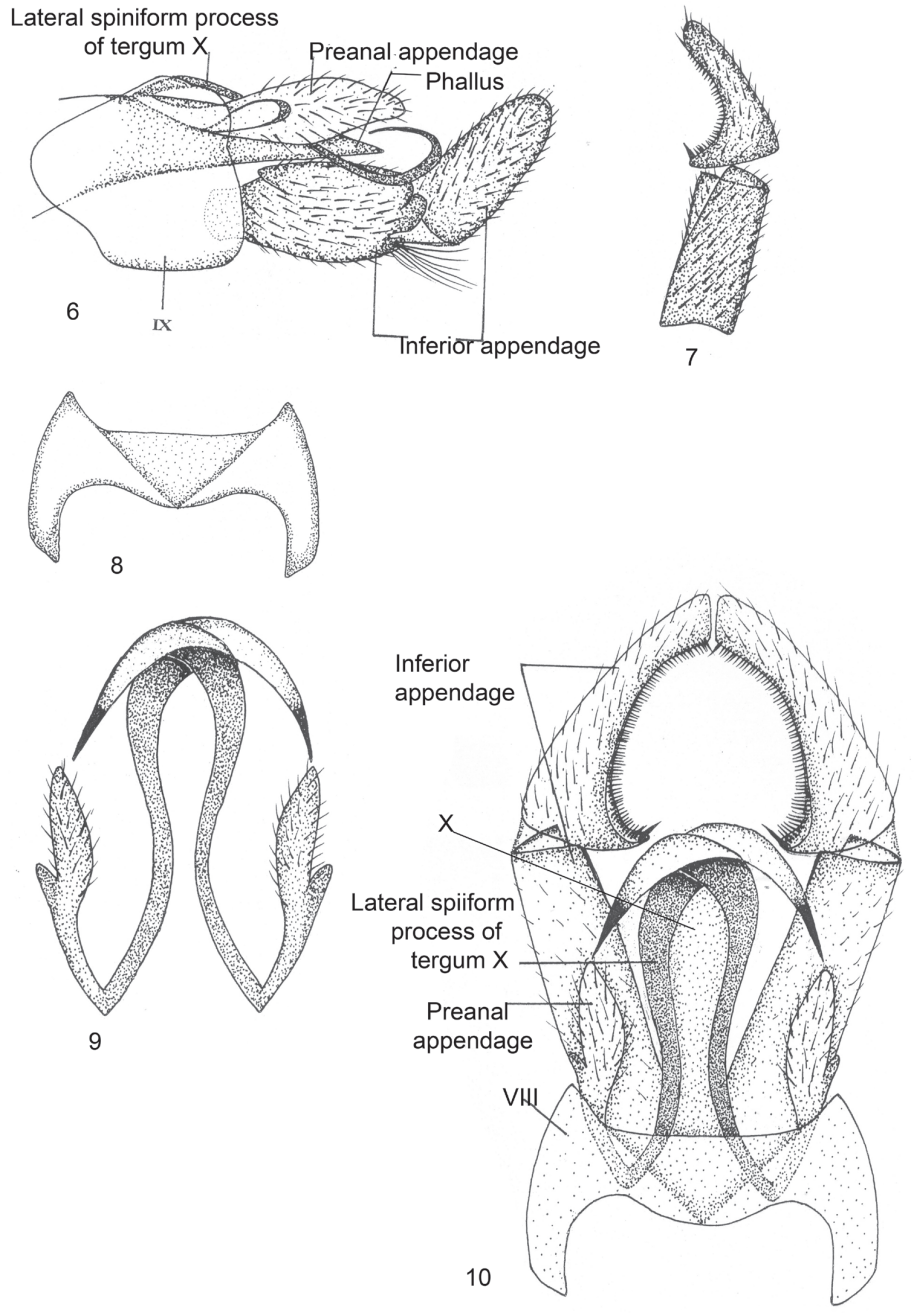
*Kisaura elongata* sp. n. **6** Male genitalia, lateral **7** Inferior appendage, dorsal **8** Tergite VIII, dorsal **9** Preanal appendages, dorsal **10** Male genitalia, dorsal

Male Genitalia (Figs 6–10). Sternum VIII without ventral process, tergum VIII not indented, posterior margin widely excised with V-shaped mark dorsally. Segment IX sclerotized, short, anterolaterally much produced, posterodorsal corner rounded. Inferior appendages long and stout, basal segment (coxopodite) as long as apical segment (harpago), basal segment with small lobe on proximal end of superior side in lateral view, and distally bilobed with inferior lobe bearing long tuft of setae; apical segment curved upright and pointed apically in lateral view, with curved row of black comb-like setae on inner surface in dorsal view. Tergum X membranous, with long inwardly recurved or bent backward, broad ribbon-like lateral spiniform processes which are pointed apically. Preanal appendages long, with two lobes of unequal size, larger lobe thumb-like while smaller wart-like in lateral view. Phallus membranous, surrounded by tergum X.

**Holotype male: INDIA: Sikkim: Gangtok:** 27°36'0"N, 88°37'0"E, 1700 m, 25.v.1999, M. Saini (PUPM).

##### Additional material.

**India: Sikkim: Rongli:** 27°13'0"N, 88°42'0"E, 900 m, 1.v.2009, Pandher and Parey, 2 males (PUPM).

##### Distribution.

India: Sikkim.

##### Etymology.

This species name pertains to excessively long lateral spiniform process of segment X of male genitalia.

#### Kisaura eloct

Pandher & Saini sp. n.

urn:lsid:zoobank.org:act:BE5FE988-64E4-4098-836F-34A49EAB5550

http://species-id.net/wiki/Kisaura_eloct

Figs 11–15 

##### Description.

This species is close to *Kisaura longispina* Kimmins 1955 in having a very long lateral spiniform process of tergum X. It also resembles *Kisaura intermedia* Kimmins 1955 in the shape of the preanal appendages. In *Kisaura eloct* sp.n. segment IX is much shorter, but wider and the basal segment (coxopodite) of the inferior appendage is long, narrow basally, and broad and truncate apically in lateral view. In *Kisaura longispina* segment X is long and somewhat quadrate posterodorsally, and the basal segment of the inferior appendage is small, but uniformly wide in lateral view. *Kisaura intermedia* differsin having the lateral spiniform process of tergum X narrow basally, broad towards its apex, and pointed apically, and also in having the superior side of the basal segment of the inferior appendage convex in lateral view whereas in *Kisaura eloct* sp. n. the lateral spiniform process of tergum X is almost uniformly wide except with a pointed apex and the basal segment is without any convexity on its superior side in lateral view.

Adult. Color in alcohol entirely fulvous, except mesoscutellum flavid; maxillary palps with pale annulations at their joints. Body covered with dense and fuscous setae except head with fulvous pubescence. Antenna long, scape:pedicel ratio= 1: 0.6; maxillary palp segments ratios 1: 2: 3: 4: 5 = 1: 1.5: 2: 1.2: 2.5 and labial palp segments ratios 1: 2: 3 = 1: 0.75: 1.75. Length of forewing 7 mm; forewing with long discoidal cell; apical fork I absent in both fore- and hind wings.

**Figures 11–15.**
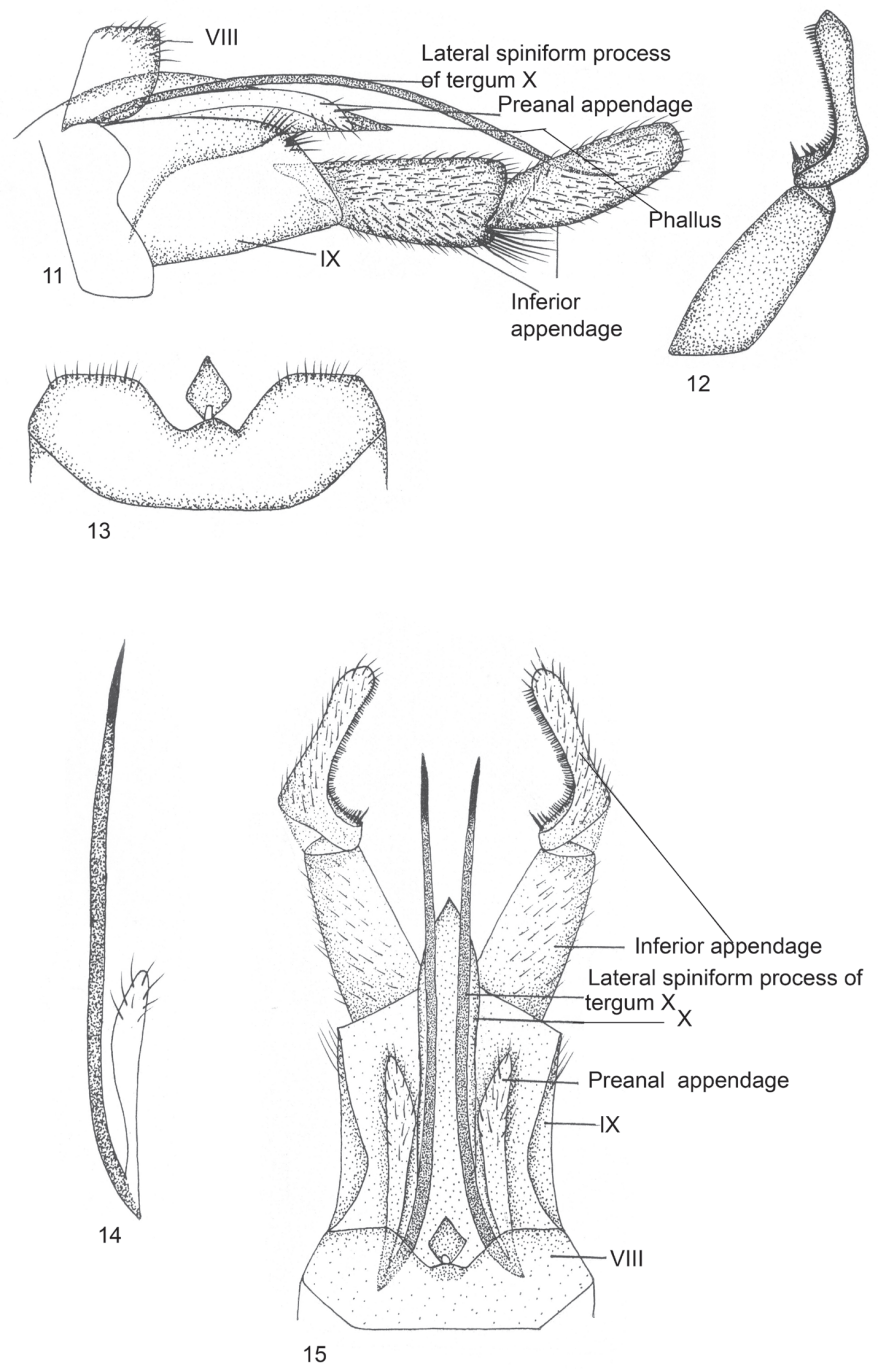
*Kisaura eloct* sp. n. **11** Male genitalia, lateral **12** Inferior appendage, dorsal **13** Tergite VIII, dorsal **14** Preanal appendage, dorsal **15** Male genitalia, dorsal

Male Genitalia (Figs 11–15).Ventral process absent on sternum VIII, tergum VIII with diamond-shaped lobe medially on dorsal surface, with two small incurved depressions on posterior margin. Segment IX sclerotized, long, trapezoidal, stout in lateral view, with posterodorsal margin slightly setose and postventrally produced apically; anterior margin covered partially by sternum VIII. Inferior appendage with basal segment (coxopodite) stout, about as long as apical, narrow at base, broad and bilobed apically in lateral view, inferior lobe with long tuft of setae; apical segment (harpago) curved upwards, with curved row of strong black brush-like setae on inner surface in dorsal view, pointed apically. Tergum X short, narrow, membranous, with pair of long lateral spiniform processes placed medially, extending almost to middle of apical segment of inferior appendage in dorsal view. Preanal appendages narrow, finger-like, scarcely dilated apically, slightly longer than segment IX in lateral view. Phallus tubular, membranous, apparently fused dorsally with membranous tergum X.

**Holotype male: INDIA: Sikkim: Gangtok:** 27°36'0"N, 88°37'0"E, 1700 m, 25.v.1999, M. Saini, (PUPM).

##### Paratypes.

same data as holotype, 2 males (PUPM).

##### Distribution.

India: Sikkim.

##### Etymology.

This species name is based on an arbitrary combination of letters (i.e., Entomology Laboratory of Caddisfly Taxonomy - “*eloct*").

#### Kisaura gangtokensis

Pandher & Saini sp. n.

urn:lsid:zoobank.org:act:38468B63-43EF-4FB9-B32C-072DF504A657

http://species-id.net/wiki/Kisaura_gangtokensis

Figs 16–9 

##### Description.

This species is close to *Kisaura rossi* Kimmins, 1955 from North East Myanmar, based on similarities in the shape of the preanal appendages in dorsal view. In *Kisaura rossi* segment IX is very short, quadrate in lateral view and the preanal appendages are not twisted at mid- length, but preapically twisted. In *Kisaura gangtokensis* sp. n. segment IX is long, trapezoidal in lateral view, and the preanal appendages are twisted at mid length.

Adult.Color in alcohol entirely fulvous and covered with moderate, fuscous setae excepting head with golden and fulvous pubescence. Antenna long, scape: pedicel ratio = 1: 0.5; maxillary palp segments ratios 1: 2: 3: 4: 5 = 1: 1.3: 2.6: 2.1: 5; labial palp segments ratios 1: 2: 3= 1: 1.5: 2. Length of forewing 7.5 mm; discoidal cell small, apical fork I lacking in both wings.

**Figures 16–19.**
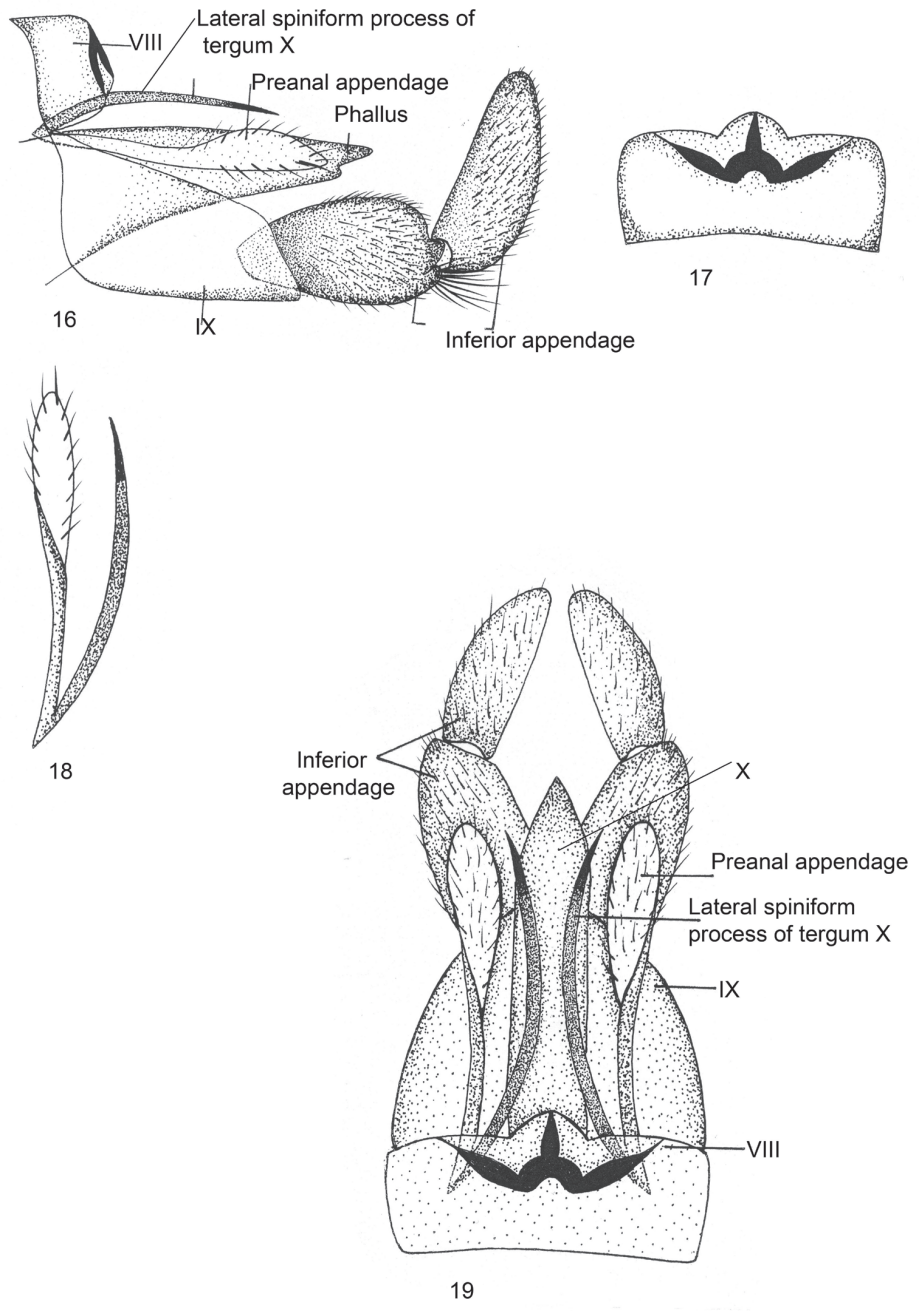
*Kisaura gangtokensis* sp. n. **16** Male genitalia, lateral **17** Tergite VIII, dorsal **18** Preanal appendage, dorsal **19** Male genitalia, dorsal.

Male genitalia (Figs 16–19).Sternum VIII without ventral process; tergum VIII roundly produced dorsomesally. Segment IXsclerotized, long, trapezoidal in lateral view, anterodorsally pointed at apex, posteroventrally much produced. Inferior appendages short, stout and two-segmented, both segments subequal in length, basal segment narrow basally, with convex superior and inferior margins, apically bilobed, inferior lobe with long tuft of setae; apical segment vertically upright, its superior margin almost straight, inferior margin convex, rounded apically, mesally with black brush-like setae. Tergum X membranous, intimately surrounding phallus, lateral spiniform process blade-like, long, almost reaching posterodorsal margin of IX in lateral view, with spinelet at apex. Preanal appendagesas long as lateral spiniform process in dorsal view twisted at mid-length, broad and rounded apically. Phallus membranous, fused dorsally with tergum X.

**Holotype male: INDIA: Sikkim: Gangtok:** 27°36'0"N, 88°37'0"E, 1700 m, 25.v.1999, M. Saini, (PUPM).

##### Paratype.

same data as holotype, 1 male (PUPM).

##### Distribution.

India: Sikkim.

##### Etymology.

This species is named after the type locality, Gangtok.

#### Kisaura himachalica

Pandher & Saini sp. n.

urn:lsid:zoobank.org:act:995FA64D-0F65-43D9-BF2F-A679AF9649D7

http://species-id.net/wiki/Kisaura_himachalica

Figs 20–24 

##### Description.

In superficial comparison *Kisaura himachalica* sp. n. is allied to *Kisaura rossi* Kimmins and *Kisaura cina* Malicky & Chantaramongkol, 1993. However *Kisaura himachalica* is distinct as segment IX is long, the basal segment of the inferior appendage is almost oval in shape, and the preanal appendage is knob-like in dorsal view. In *Kisaura rossi* segment IX is small, the basal segment of the inferior appendage is quadrate, and thr preanal appendage is twisted preapically and not knob-shaped. The new species also differs in the shape of segment IX, which is broader, and the preanal appendages, which are bilobed and clavate in dorsal view in *Kisaura cina*.

Adult. Color in alcohol uniformly fulvous and body covered with inconspicuous, sparse and fulvous setae excepting mesoscutellum where is golden. Antenna long, scape:pedicel ratio = 1: 0.7; maxillary palp segments ratios 1: 2: 3: 4: 5 = 1: 1.5: 2.5: 2: 5; labial palp segments ratios 1: 2: 3= 1:1.2: 1.75. Forewing with golden irrorations; length of forewing 7 mm, dicoidal cell small, apical fork I lacking in both wings.

**Figures 20–24.**
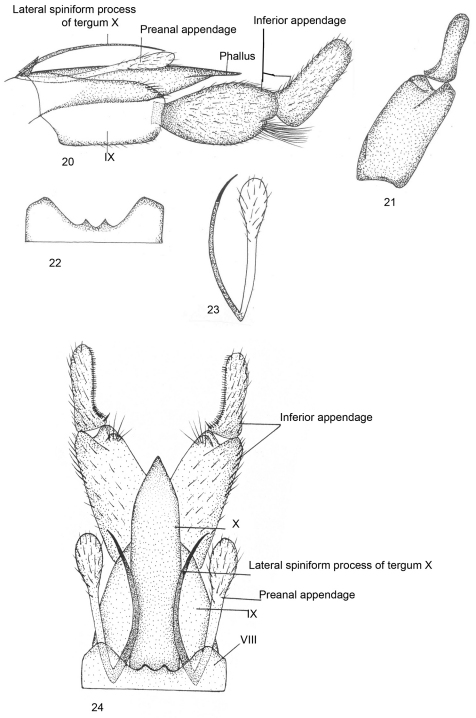
*Kisaura himachalica* sp. n. **20** Male genitalia, lateral **21** Inferior appendage, dorsal **22** Tergite VIII, dorsal **23** Preanal appendage, dorsal **24** Male genitalia, dorsal

Male genitalia (Figs 20–24).Ventral process absent on sternum VIII; tergum VIII with apical margin produced into two small triangular extensions, separated by shallow excision, area between the two lobes slightly elevated. Segment IX roughly quadrate, anterodorsally pointed, anteromedially with excision, posterodorsally rounded. Inferior appendage with basal segment equal in length to apical segment but stouter than the later, roughly oval in shape, superior and inferior sides convex, divided into two lobes, inferior one with path of long setae, apical segment slender, incurved, with curved row of stout black comb-like setae on inner surface in dorsal view. Tergum X membranous with pair of sword-like lateral spiniform process, about as long as segment IX. Preanal appendage as long as segment IX, clavate in lateral view, apically bulbous and setose in dorsal view. Phallus tubular, membranous, apparently surrounded by Tergum X.

**Holotype male: Himachal Pradesh: Barot:** 32°17'0"N, 75°77'0"E, 2300 m, 22.v.1998, M. Saini, (PUPM).

##### Distribution.

India, Himachal Pradesh.

##### Etymology.

This species is named after the state in which the holotype was found, Himachal Pradesh.

#### Kisaura truncata

Pandher & Saini sp. n.

urn:lsid:zoobank.org:act:35D84A5D-ADFC-4BD4-AE82-865821D1DC42

http://species-id.net/wiki/Kisaura_truncata

Figs 25–29 

##### Description.

This species resembles *Kisaura gangtokensis* sp. n.,based on the ratio of the lengths of the lateral spiniform process and preanal appendages, but differs in several aspects: the preanal appendages are long and slender, segment IX is broad anterolaterally and without rounded lobes at the posterolateral end, and the basal segment of the inferior appendage is long and the apical segment is directed upright and truncate apically.

Adult. Color in alcohol entirely fulvous excepting fuscous mesoscutellum. Antenna almost 1.2 × longer than forewing, scape:pedicel ratio = 1: 0.46; maxillary palp segments ratios 1: 2: 3: 4: 5= 1: 2.6: 3.3: 2.6: 5.6; labial palp segments ratios 1: 2: 3= 1: 1: 2. Forewing with white spots and covered with moderate and short setae; length of forewing 9 mm; discoidal cell small, triangular; apical fork I absent in both wings.

**Figures 25–29.**
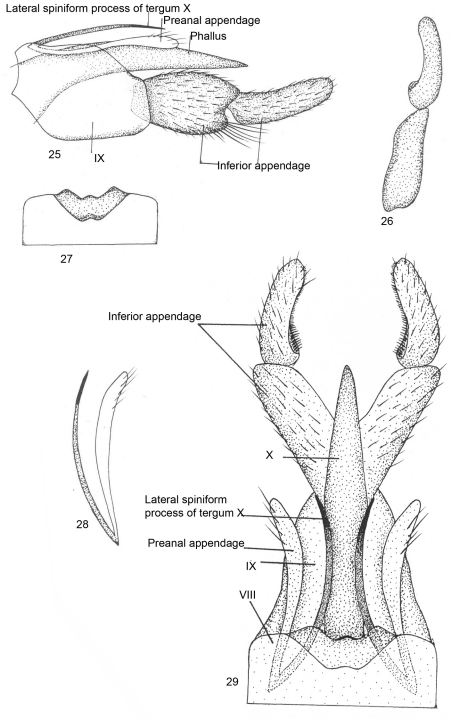
*Kisaura truncata* sp. n. **25** Male genitalia, lateral **26** Inferior appendage, dorsal **27** Tergite VIII, dorsal **28** Preanal appendage, dorsal **29** Male genitalia, dorsal.

Male genitalia (Figs 25–29). Sternite VIII without ventral process, tergite VIII indented dorsomesally, with rounded median lobe. Segment IX long and broad, roughly pentagonal, anterodorsally with pointed median prominence in lateral view. Inferior appendage two-segmented, basal segment convex on lateral margin, broader, bilobed at apex with short inferior lobe and bearing tuft of long setae; apical segment nearly as long as basal segment, narrower, with curved row of spines, rounded at apex in dorsal view. Tergite X membranous, almost reaching apex of basal segment of inferior appendage, with lateral spiniform processes extending beyond apical margin of segment IX, with spinelet at apex. Preanal appendage almost equal to lateral spiniform process, rounded apically in lateral view. Phallus membranous and fused with tergite X.

**Holotype male: INDIA: Sikkim: Gangtok:** 27°36'0"N, 88°37'0"E, 1700 m, 25.v.1999, M.Saini, (PUPM).

##### Additional material.

 **INDIA: Sikkim: Singhik:** 27°31'0"N, 88°34'0"E, 1900 m, 14.ix.2009, Pandher, 2 males (PUPM).

##### Distribution.

India: Sikkim.

##### Etymology.

This species is named for the truncate posterolateral margin of segment IX.

## Supplementary Material

XML Treatment for Kisaura

XML Treatment for Kisaura clavata

XML Treatment for Kisaura elongata

XML Treatment for Kisaura eloct

XML Treatment for Kisaura gangtokensis

XML Treatment for Kisaura himachalica

XML Treatment for Kisaura truncata
